# Electroacupuncture at Acupoint ST36 (Zusanli) Improves Intestinal Motility Dysfunction Via Increasing the Proportion of Cholinergic Neurons in Rat Ileal Myenteric Ganglia after Severe Acute Pancreatitis

**DOI:** 10.1155/2022/7837711

**Published:** 2022-10-22

**Authors:** Xueling Wang, Lingyun Lu, Liu Zi, Hangqi Hu, Hao Li, Ying He, Ning Li, Qian Wen

**Affiliations:** Integrated Chinese and Western Medicine Department, West China Hospital, Sichuan University, Guoxue Lane No. 37, Wuhou District, Chengdu, Sichuan 610041, China

## Abstract

Using a severe acute pancreatitis (SAP) rat model, the mechanism of electroacupuncture (EA) were studied on the intestinal function of pancreatitis. The SAP models were established by injecting 30% L-ornithine at hourly intervals, and were divided into two groups (14 in each): SAP model group, which was not treated, and EA group, which received EA at ST36 at a frequency of 1-2 Hz and amplitude of 1 mA for 30 min twice a day. Fourteen rats were also included as the control group. After EA, the intestinal propulsion was measured. In the distal ileum myenteric plexus, the density of HuC/D and the proportion of cholinergic neurons were measured using immunohistochemistry. Compared to the SAP model group, the EA group demonstrated significant improvements in intestinal propulsion rates. Furthermore, after EA, the density of myenteric neurons in the ileum returned to normal levels and the proportion of cholinergic neurons was increased compared to the SAP model group. And finally, EA alleviated the damage to the pancreas. Thus, our results suggest that EA stimulation at ST36 can partly restore the enteric neuron function and improve intestinal motility dysfunction, therefore could ameliorate SAP. The enteric nervous system can participate in changes in intestinal motility by affecting cholinergic neurons.

## 1. Introduction

Acute pancreatitis (AP) is a common gastrointestinal (GI) disease with acute inflammatory edema and necrosis of the pancreas, causing major morbidity and mortality [1]. According to a recent report, approximately 34 out of 100,000 people are afflicted with AP every year, and the incidence is increasing worldwide [2]. AP is highly variable, ranging from mild to modest, even severe, which is associated with several complications and organ failure. Severe acute pancreatitis (SAP) is one of the most life-threatening forms of pancreatitis responsible for the mortality of approximately 5–42% of cases. It demonstrates a decreasing trend depending on the country and the quality of the intensive care [3–6]. However, it is still a heavy social burden in the US [7]. Evidence has shown that mortality due to SAP with pancreatic infection is higher than that of SAP without infection [8]. Bacterial translocation and infection are presumed to be responsible for the aggravation of AP [9], and the mechanism of translocation may be due to intestinal dysmotility [10]. Clinical studies also showed that SAP patients demonstrate GI disorders such as abdominal pain and distension, paralytic intestinal obstruction, and cessation of defecation [11, 12]. Moreover, improving GI dysfunction proves to be beneficial to the AP patients [13]. All of these indicate the importance of gastroenteric function in AP.

As a significant part of Chinese medicine, acupuncture has been proven to be effective for treating pancreatitis. A clinical randomized controlled trial showed that acupuncture significantly reduces the abdominal distension in patients with SAP with paralytic ileus, proving the benefits of acupuncture for treating SAP [14]. Animal studies have also shown that acupuncture at ST36 could modulate the inflammatory mediators, and alleviate serum inflammatory factors in the SAP rat model, indicating that acupuncture is effective against SAP owing to its anti-inflammatory properties [15]. Furthermore, Guo's report demonstrated that EA at ST25 and ST36 could promote GI motility in rats with SAP [16]. Thus, EA has the ability to regulate GI motility in SAP. However, the mechanism through which EA improves GI motility in SAP is not very clear.

The enteric nervous system (ENS) is an independent neuronal network, which is widely distributed throughout the intestinal tract, composed of myenteric and submucosal plexuses and necessary for ordinary GI motility [17]. The basis of propulsive motility patterns is peristaltic reflexes involving excitatory motor neurons, inhibitory motor neurons, and sensory neurons [18]. An alteration in the ENS has been recognized in some diseases, such as irritable bowel syndrome [19], slow transit constipation [20], pancreatitis [21], diabetes [22], and Parkinson's disease [23]. The evidences of EA effect to nervous system, even ENS has been proved [24]. EA at ST36 has been proved to initiate through special PROKR2Cre-marked sensory neurons to activate the vagal–adrenal axis [25]. Besides, EA improved gastric motility by increased the expression level of choline acetyltransferase (ChAT) proteins, and ChAT mRNA in the ENS of diabetic rats [26]. Given that EA can have a major influence on the nervous system, it was hypothesized that EA could modulate the ENS of SAP to rescue the GI dysmotility. Therefore, in this study, we established the L-ornithine-induced pancreatitis model in rats in order to examine the changes in ChAT-immunoreactive (ChAT-IR) neurons in myenteric ganglia.

## 2. Methods

### 2.1. Animals and Experimental Models

Male Sprague-Dawley rats (180–220 g), from Chengdu Dashuo Experimental Animal Co. Ltd (Chengdu, China) were raised in standard animal house conditions, including an SPF animal facility under a 12-h light/dark cycle, 23 ± 2°C, and humidity of 40–70%. All animal care and experimental protocols were approved by the Ethics Committee of West China Hospital of Sichuan University (Approval number: 2020002A). All the rats were acclimatized for 7 days and subjected to fasting for 12 h before the experiments. The rats were randomly divided into three groups: control group (*n* = 14), SAP model group (*n* = 14) and EA group (*n* = 14). SAP was induced by intraperitoneal injection of 30% L-ornithine dissolved in saline (3.0 g kg^−1^, pH = 7.4) at hourly intervals. The control rats were injected with vehicle solution (saline). All the rats were subjected to 3 times oral gavage of saline (2 mL 100 g^−1^) at intervals of 2 h after the last injection to rehydrate after being devoid of food for 24 h.

### 2.2. EA Intervention

The EA group rats were bound with a special apparatus, which could fix the rat in a comfortable position with a plastic tube and expose the hind limbs to prevent slipping and turning (the details of apparatus was showed on Supplemental material). Bilateral ST36 were pierced with acupuncture needles of type 0.23 mm × 13 mm, and stimulated by SDZ-V-type EA treatment instrument (1-2 Hz, 1 mA) for 30 min (ST36 is located 5 mm below the lower border of the patella, 1.5 mm width lateral from the anterior border of the tibia of rat according to the “Animal acupuncture points diagram,” made by experimental research association of China acupuncture-moxibustion science). Since “De Qi,” an essential component for acupuncture efficacy, is not known in animals, the EA frequency is appropriate for slight shaking of the lower extremity. Acupuncture treatments were applied twice: immediately following SAP induction and an hour before sacrifice. Rats in the control and SAP groups were restrained in the same way but did not receive any treatment.

### 2.3. Assessment of the Small Intestinal Transit

Trypan blue (1 g; Sigma, St. Louis, MO, USA) was diluted in 100 mL double-distilled water, and 1.5 mL of the solution was administered into the stomach by gavage immediately after 24 h from the last injection. The rats were sacrificed 30 min later for measurement of the small intestinal transit.

### 2.4. Histopathologic Analysis

After 24 h of modeling, rats in all groups were anesthetized with amytal administered intraperitoneally (0.8 g kg^−1^). Pancreas were dissected and fixed overnight at room temperature in a pH-neutral, phosphate-buffered, 10% formaldehyde solution. The tissue was then embedded in paraffin, sectioned at a thickness of 4 *μ*m, and stained with hematoxylin and eosin (HE). The severity of pancreatic damage was graded according to Schmidt's report [27]. Briefly, HE-stained sections were graded in a blinded fashion for the extent and severity of edema (0–4), hemorrhage and fat necrosis (0–4), inflammatory infiltration (0–4), and acinar necrosis (0–4). The scores for each histopathologic parameter were summed up, leading to a minimum score of 0 and a maximal possible score of 16.

### 2.5. Immunohistochemistry

The tissue sections (5 *μ*m) were incubated overnight at room temperature with primary antibodies against pan-neuronal HuC/D and then exposed to biotinylated immunoglobulins, peroxidase-labeled streptavidin complex, and 3, 3′-diaminobenzidine tetrahydrochloride. Three sections for each ileal sample (*n* = 6 animals per group) were examined under a light microscope, and representative photomicrographs were assessed for quantitative evaluation. Neuronal density was estimated as the number of HuC/D-immunostained cells within the ganglionic area [28]. To measure the myenteric ganglionic area, morphometric analysis was carried out on at least 10 microscopic fields among all sections, captured with a 40x objective, and the area of the ganglia was expressed in mm^2^ calculated by Image-Pro Plus 6.0.

### 2.6. Double Immunofluorescence

From each rat, a 1.0 cm segment of the distal ileum was collected. The specimen was subsequently fixed in 10% paraformaldehyde, pH 7.2 for 4 h at room temperature and overnight in a solution of 30% sucrose at 4°C. The whole-mount preparation of the myenteric ganglia was prepared by peeling off the circular smooth muscle layer with fine forceps. The double-immunolabeling study was done by incubating the specimens in 10% normal goat serum and 0.5% Triton X-100 in PBS for an hour at 37°C to block non-specific binding of antibodies and enhance penetration of the antibody. The indirect immunofluorescence method was used for double-immunolabeling.  Table 1 summarizes the antibodies used and their dilutions. In this study, anti-HuC/D antibodies were used as a general neuronal marker. Tissue was incubated with primary antibodies at 37°C in a humid chamber for 24 h. Each primary antiserum was applied separately. After washing in PBS (5 × 5 min), the tissue was incubated for one hour at room temperature in a humid chamber, with secondary antibodies of tetramethylrhodamine isothiocyanate (TRITC)-labeled goat anti-rabbit-IgG and fluorescein isothiocyanate (FITC)-labeled goat anti-mouse-IgG. After washing in PBS (5 × 5 min), the tissue was mounted on glass slides using glycerol and examined under a fluorescence microscope (Zeiss, Axiovert 200, Germany). To estimate the proportion of ChAT-IR neuron of myenteric ganglia, at least 200 neurons were calculated in 5–10 microscopic fields randomly selected and the proportion was expressed as the percentage of HuC/D immunoreactive neurons.

### 2.7. Data Analysis

Data from all the experiments are expressed as mean ± standard error values. Multiple group comparisons were conducted using one-way ANOVA. All the data analyses were performed using GraphPad Prism, version 8.0.1 (GraphPad Software Inc., San Diego, CA, USA). *P* < 0.05 was considered to indicate statistical significance.

## 3. Results

### 3.1. Effect of EA on Small Intestine Propulsion

**Figure 1:**
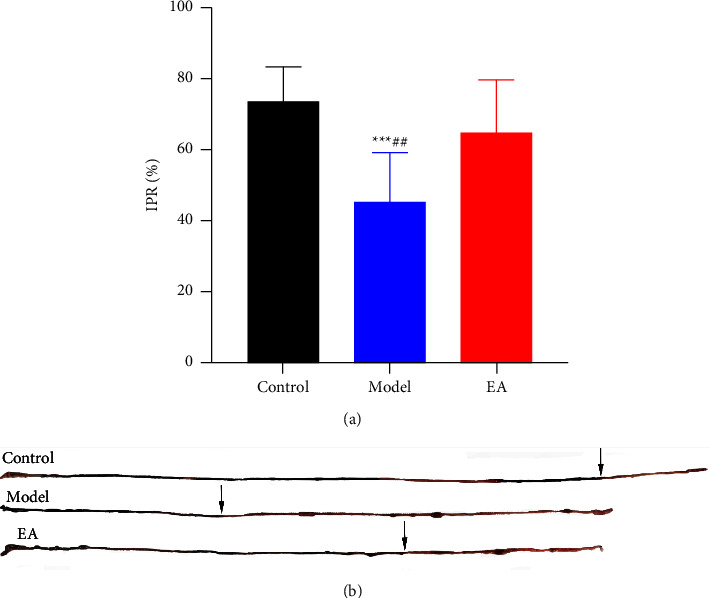
Effect of electroacupuncture on the intestinal propulsion rate. All groups were used in the intestinal propulsion rate. Example of the intestine and trypan blue trace for all groups. ((a and b) *n* = 8 for each group). ^∗∗∗^*P* < 0.001 vs. control group, ^##^*P* < 0.01 vs. EA group.

Trypan blue intestinal propulsion experiments showed the distances by which trypan blue propulsion decreased in SAP models compared with that in the control group, this alternation was considered to be significant (*P*=0.0003 vs. Model group, Figures 1(a) and 1(b)). After EA at ST36, the intestinal propulsion rates (IPR) significantly increased compared to the model group (*P*=0.0075 vs. Model group, Figures 1(a) and 1(b)). Although the EA stimulation made a resume to the IPR, it did not return to values similar to the control group with a no significant decreased tendency (*P*=0.1704 vs. Control group, Figure 1(a)).

### 3.2. Effect of EA on the Density of Neurons of Intestinal Myenteric Plexus

**Figure 2:**
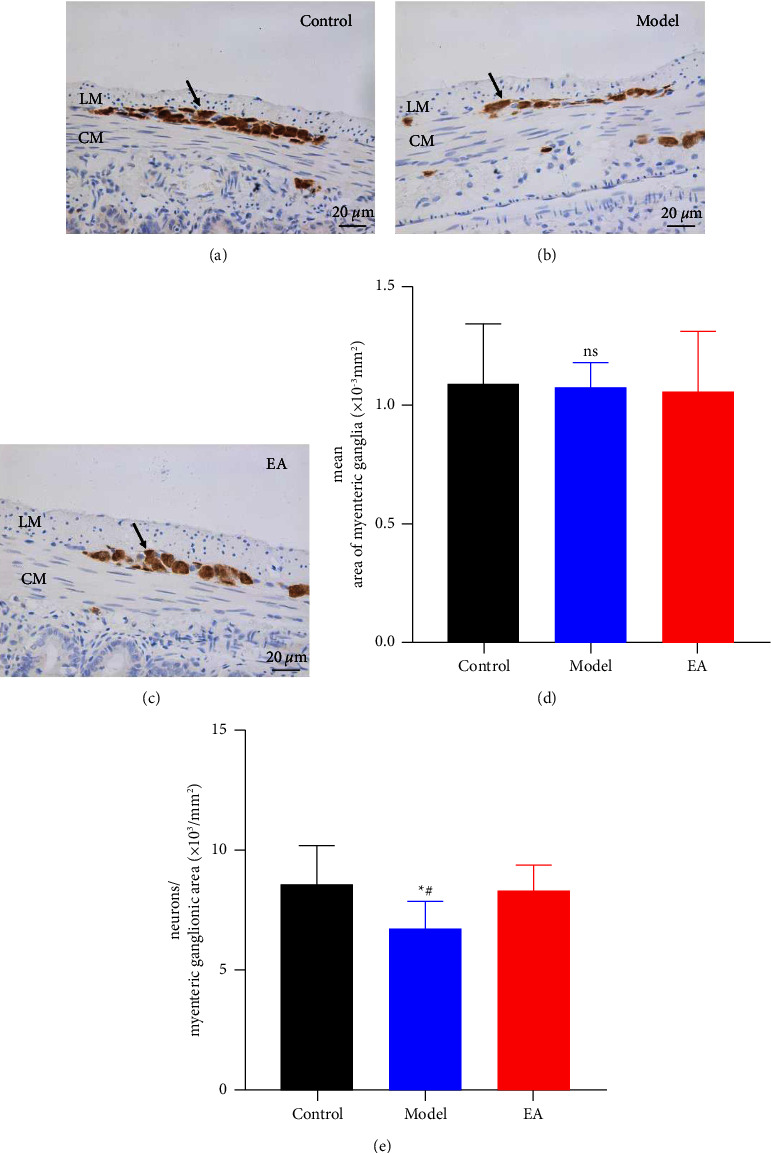
Effect of electroacupuncture on the density of neurons of the myenteric ganglia. Tissue with brown granular deposits indicates positive HuC/D immunostaining (arrows). Scale bar: 20 um, magnification: ×400. The distal ileum (a–c). The area of myenteric ganglia (d). The mean number of HuC/D immunoreactive myenteric neurons (e) *n* = 6 for each group). ^∗^*P* < 0.05 vs. control group, ^#^*P* < 0.05 vs. EA group. LM: longitudinal muscle. CM: circular muscle.

To explore the mechanism of EA increasing IPR, we assessed the density of myenteric neurons, the area of myenteric ganglia was quantitatively measured, and no significant changes were found among the three groups (Figure 2(d)). Immunohistochemical staining revealed that in the ileum, the density of the HuC/D immunoreactivity neurons in the SAP model was markedly decreased compared to that in the control group (*P*=0.0233, Figures 2(b) and 2(e)). After EA treatment, the density of HuC/D was significantly increased compared to SAP model and was similar to that in the control group (*P*=/0.0479 vs. Model group, *P*=0.7155 vs. Control group, Figures 2(c) and 2(e)).

### 3.3. Effect of EA on the Proportion of ChAT-IR Neurons

**Figure 3:**
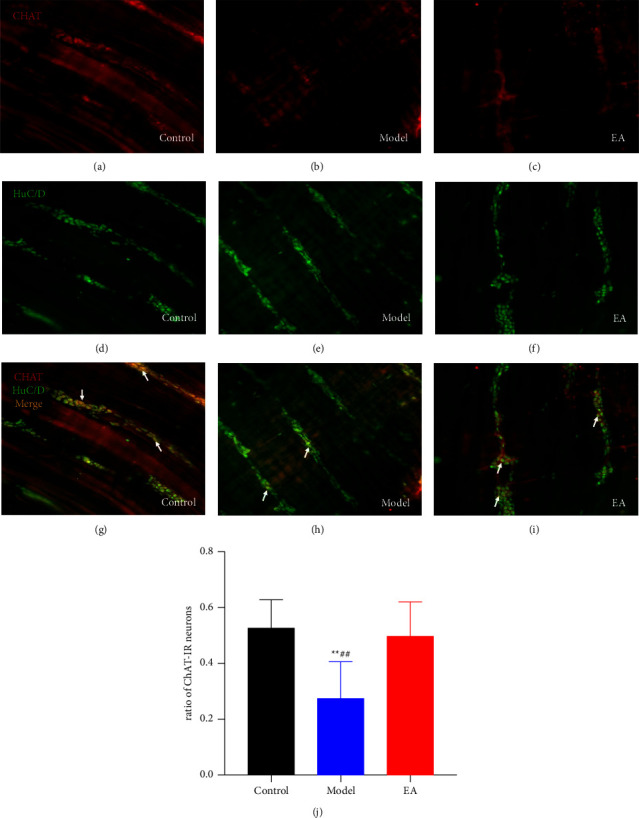
Effect of electroacupuncture on the proportion of ChAT-IR neurons. Double labeling for HuC/D (green), ChAT (red), and merge (yellow, arrows) of whole-mount preparations of myenteric ganglia. Magnification: ×200 (a–i). The ratio of ChAT-IR immunoreactive myenteric neurons (j) *n* = 6 for each group). ^∗∗^*P* < 0.01 vs. control group, ^##^*P* < 0.01 vs. EA group.

The double immunofluorescence of the whole-mount preparation of the ileum was used to calculate the proportion of ChAT-IR neurons (Figures 3(a)–3(i)). In each group (*n* = 6), more than 200 myenteric neurons were evaluated in 10 random fields. In the SAP group, the statistical analysis showed that the percentage of ChAT-IR neurons was significantly lower compared to that in the control group (*P*=0.0022, Figure 3(j)). After the ST36 stimulation, the proportion of ChAT-IR neurons was increased significantly in the EA group compared to that in the SAP (*P*=0.0063 vs. Model group), such that this group did not differ remarkably from the control group (*P*=0.6195 vs. Control group).

### 3.4. Histological Examination of Pancreas Treated with EA

**Figure 4:**
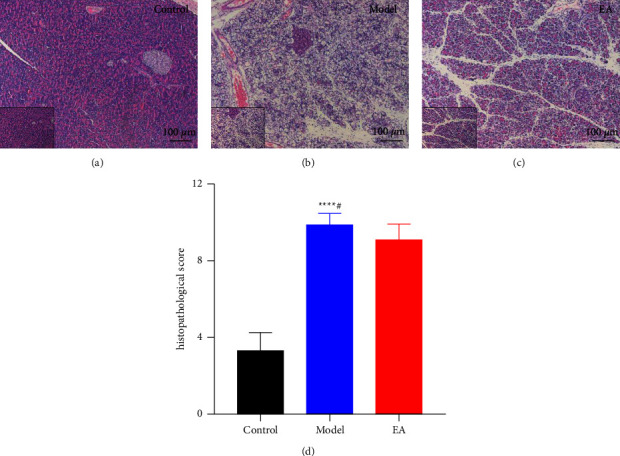
Effect on histopathological examinations of the pancreas. Scale bar: 50 *µ*m, magnification: ×200. The pancreatic edema, necrosis, and inflammatory infiltration were showed by all groups' H&E staining (a–c). Histopathological score of the pancreas (d) *n* = 8 for each group). ^∗∗∗∗^*P* < 0.0001 vs. control group, ^#^*P* < 0.05 vs. model group.

We finally used the H&E staining to determine if the electroacupuncture at ST36 could improve the impairment of pancreas by alter the intestinal dysfunction. Administration of intraperitoneal L-ornithine injections every hour resulted in typical features of SAP, characterized by severe interstitial edema, diffuse pancreatic acinar cell necrosis, extensive inflammatory cell infiltration, and hemorrhage compared to the control group (Figures 4(a) and 4(b)). After EA treatment, the pathological lesion of the pancreas was significantly decreased (Figure 4(c)) and the histopathological scores were significantly different from those of the SAP group (*P*=0.0272 vs. Model group, Figure 4(d)), while it was significantly increased compared to the control group (*P* < 0.0001 vs. Control group, Figure 4(d)).

## 4. Discussion

Using intestinal propulsion experiments, our study demonstrated EA at ST36 to be effective in treating intestinal dysmotility associated with the L-ornithine SAP rat model. Immunohistochemistry of HuC/D showed that impaired myenteric neurons in the SAP group could be rescued by EA treatment. Double-immunolabeling of HuC/D and ChAT showed that the percentage of ChAT-IR neurons in the EA group was significantly higher than that in the SAP group. As ChAT is a recognized maker of cholinergic neuron, all these findings strongly suggest that intestinal dysmotility in L-ornithine SAP rats are related to the damage of acetylcholine (Ach) neurons in the ileal myenteric ganglia, and the traditional Chinese therapy, EA, could ameliorate this impairment.

Acupuncture is an alternative treatment that is effective against GI functional disorders, including ileus, constipation, irritable bowel syndrome, and intestinal dysmotility-associated pancreatitis [29–31]. In this study, the results of the small intestinal transit experiment indicated that EA at ST36 improves impaired intestinal function, accelerating intestinal motility. ST36 is on the stomach meridian, located in the hind limb, and clinical studies have demonstrated that acupuncture at the hind limb increases GI motility [32]. Previous studies have proved that EA at ST36 may activate the cholinergic anti-inflammatory pathway, and alleviate the inflammation in pancreatitis [15]. In addition, researches have shown that EA stimulation of abdominal points influences the sympathetic nerves, while points in the four limbs influence the parasympathetic nerves [24]. For GI motility, the parasympathetic nerves can promote peristalsis, while sympathetic nerves inhibit it [33]. Accordingly, stimulation at ST36 could not only rescue the inflammation of the pancreas but also improve GI motility disorders associated with pancreatitis by stimulating the parasympathetic nerves. However, the underlying mechanisms of EA at ST36 on the ileus of SAP have not been well studied.

The ENS is a complex network of neurons and glia that reside in the myenteric and submucosal plexus of the GI and it controls bowel function, including peristalsis [34, 35]. The ENS mechanisms associated with pancreatitis-induced intestinal dysmotility have been previously demonstrated. Zhou's study with L-ornithine SAP rats showed that the small intestinal paralysis, indicated by the small intestine electrical activity, is related to the deficiencies in ICC and nNOS neurons [36]. Another study indicated that the same neurons and cholinergic neurons are impaired with SAP at 24 h, and could be improved by octreotide [10]. However, Lin and colleagues reported contrasting results that bile duct perfusion-induced rat pancreatitis model showed an upregulation of nNOS myenteric neurons without a decrease in the HuC/HuD neurons, and they suggested that different SAP models induce different changes in myenteric neurons [21]. In Rakonczay's rat models of L-ornithine-induced ANP, dilated small bowel were observed apparently at 72 h after L-ornithine administration [37]. All of these findings support an underlying neuron injury mechanism of GI dysfunction in L-ornithine-induced SAP. In the ENS, ACh is a major excitatory neurotransmitter that regulates intestinal motility and is synthesized by the cytoplasmic enzyme choline acetyltransferase (ChAT) [38]. ChAT can mark the peripheral cholinergic neurons, making it possible to identify cholinergic neurons in the ENS [39]. Furthermore, HuC/D is a neuron-specific protein distributed in the cytoplasm and nucleus, commonly used as a pan-neuronal marker [40]. Therefore, our immunohistochemistry of HuC/D in the bowel layers showed the reduction in density of neurons, and double immunofluorescence showed a decreased proportion of ACh neurons. Thus, the deficiency of ACh in the myenteric plexus might reduce intestinal motility, and be closely related to the development of intestinal paralysis associated with SAP.

In our study, the immunohistochemistry results showed the density of ileal myenteric neurons were significantly increased after EA ST36 treatment compared to SAP models, indicating that EA may improve enteric neurons and repair the impaired ENS to restore the GI dysmotility with SAP. But what made us noteworthy was the *P* value of neuron density between two groups, it was close to 0.05, which means we need more sample sizes and methods to confirm the change of neuron. Previous studies have found that EA at ST36 could induce regeneration of lost enteric neurons in the diabetic model, and ST37 could ameliorate enteric neuron impairment in the constipation model [41, 42]. Thus, in combination with our results, we suggest that EA ST36 stimulation could affect the intestinal motility through cholinergic neurons in ENS in the SAP model. In our opinion, EA may improve pancreatitis via repair of the ENS to avoid further infection and to reduce inflammation; the pathological results of the pancreas support this hypothesis. Recent studies have established that EA at ST25 and ST37 could modulate excitatory and inhibitory neurons in the enteric nervous system to affect the colonic motility [43]. ACh is a major excitatory neurotransmitter synthesized by the cholinergic neurons. Moreover, Zhou's report had shown the reduction of ACh neurons in myenteric plexus in the SAP model [36]. Similarly, this study found significantly reduced expression of ACh neurons in the SAP model compared with that in the control group, and a rescue of this phenotype by EA treatment at ST36. Thus, we speculated that EA at ST36 could restore the impaired ENS function, resulting in up-regulated levels of neurotransmitters in the enteric excitatory motor neurons and thereby ameliorating pancreatitis.

However, there are still many limitations should be considered. First, according to our research data, it is hard to confirm if the beneficial effect of electroacupuncture is mediated locally or systemically, and find out the causality between inflammation and motility. Although we did a useful exploration, we will need more investigations. Second, other excitatory and inhibitory neurons were not investigated in the present study. As their functions are various, it is difficult to examine if these neurons are related to the GI motility in SAP; further studies are required to confirm their association with acupuncture. Third, SAP is a disease with poor prognosis, we still wonder if the acupuncture could improve the prognosis such as the mortality and will need to be determined.

## 5. Conclusions

In summary, EA stimulation at ST36 is capable of restoring the GI dysmotility in the L-ornithine SAP model by modulating the ENS, especially the ACh neurons. EA could ameliorate pancreatitis through the central and peripheral neural pathways, working in concert with the ENS.

## Figures and Tables

**Table 1:** List of antibodies used in this study.

Antibody	Species	Code	Dilution	Source
*Primary antibodies*				
HuC/D	Mouse	A-21271	1 : 100	Molecular probes; Eugene, OR
ChAT	Rabbit	A13244	1 : 100	ABclonal; Wuhan, CHN

*Secondary antibodies*				
FITC—conjugated goat anti-mouse IgG	Goat	A0568	1 : 300	Beyotime biotechnology; Shanghai, CHN
TRITC—conjugated goat anti-rabbit IgG	Goat	511202	1 : 300	Zen bioscience; Chengdu; CHN
Labelled polymer—dako real EnVision-HRP	Rabbit-mouse	K5007		Agilent technologies; California; US

## Data Availability

The data used to support the findings of this study are available from the corresponding author upon request.
